# Entwicklung und Evaluation einer Ultraschallnavigation für Freihandbiopsien kleiner Raumforderungen im Kopf-Hals-Bereich

**DOI:** 10.1007/s00106-023-01385-9

**Published:** 2023-12-05

**Authors:** Claudia Scherl, Marie Otto, Ibrahim Ghanem, Javier Moviglia, Fabian Sadi, Tirza Gnilka, Nicole Rotter, Lena Zaubitzer, Jan Stallkamp

**Affiliations:** 1 Klinik für Hals-Nasen-Ohrenheilkunde, Kopf- und Halschirurgie, Medizinische Fakultät Mannheim, Universität Heidelberg, Theodor-Kutzer-Ufer 1–3, 68167 Mannheim, Deutschland; 2 AI Health Innovation Cluster, Heidelberg-Mannheim Health and Life Science Alliance, Heidelberg, Deutschland; 3 Mannheim Institute for Intelligent Systems in Medicine (MIISM), Medizinische Fakultät Mannheim, Universität Heidelberg, Heidelberg, Deutschland

**Keywords:** Akustisches Feedback, Tracking, Phantom, Tiefes neuronales Netz, Acoustic feedback, Tracking, Phantom, Deep neural network

## Abstract

**Hintergrund:**

Der Ultraschall als wichtiges Bildgebungsverfahren im Kopf-Hals-Bereich ist leicht verfügbar, dynamisch, kostengünstig und ohne Strahlenbelastung. Eingriffe in der komplexen Kopf-Hals-Anatomie erfordern eine gute Orientierung, die durch Navigationssysteme unterstützt wird.

**Ziel der Arbeit:**

Entwicklung eines neuen ultraschallkontrollierten Navigationssystems zur Punktion kleiner Zielstrukturen im Kopf-Hals-Bereich.

**Methodik:**

Es wurde ein Halsphantom mit sonographierbaren Raumforderungen (RF; Größe: 8–10 mm) konstruiert. Diese wurden automatisch mittels eines ResNet-50-basierten tiefen neuronalen Netzes segmentiert. Der Ultraschallkopf (UK) wurde mit einem individuell hergestellten Trackingtool versehen.

**Ergebnisse:**

Die Positionen von Ultraschallgerät, RF und Punktionsnadel wurden im Weltkoordinatensystem erfasst. In 8 von 10 Fällen wurde eine 8 mm große RF getroffen. Die durchschnittliche Abweichung wurde mit 2,5 mm in einem speziellen Evaluationsphantom berechnet. Die getrackte Biopsienadel wird durch auditives Feedback ausgerichtet und zur RF navigiert.

**Schlussfolgerung:**

Herausragende Vorteile im Vergleich zu herkömmlichen Navigationssystemen sind: Verzicht auf präoperative Schnittbildgebung, automatische dreidimensionale Echtzeitregistrierung, welche die intraoperative Gewebeverschiebungen berücksichtigt, Beibehaltung der optischen Achse des Operateurs auf den Situs, ohne dass auf einen Navigationsmonitor geschaut werden muss, und beidhändiges Arbeiten ohne Halten des UK während der Punktion. Insgesamt lässt sich das beschriebene Funktionsmuster außer für Nadelbiopsien auch in der offenen Kopf-Hals-Chirurgie anwenden.

**Zusatzmaterial online:**

Zusätzliche Informationen sind in der Online-Version dieses Artikels (10.1007/s00106-023-01385-9) enthalten.

## Abstract (en)

**Background:**

Ultrasound is an important imaging method in the head and neck area. It is readily available, dynamic, inexpensive, and does not involve radiation exposure. Interventions in the complex head and neck anatomy require good orientation, which is supported by navigation systems.

**Objective:**

This work aimed to develop a new ultrasound-controlled navigation system for taking biopsies of small target structures in the head and neck region.

**Methods:**

A neck phantom with sonographically detectable masses (size: 8–10 mm) was constructed. These were automatically segmented using a ResNet-50-based deep neural network. The ultrasound scanner was equipped with an individually manufactured tracking tool.

**Results:**

The positions of the ultrasound device, the masses, and a puncture needle were recorded in the world coordinate system. In 8 out of 10 cases, an 8‑mm mass was hit. In a special evaluation phantom, the average deviation was calculated to be 2.5 mm. The tracked biopsy needle is aligned and navigated to the masses by auditory feedback.

**Conclusion:**

Outstanding advantages compared to conventional navigation systems include renunciation of preoperative tomographic imaging, automatic three-dimensional real-time registration that considers intraoperative tissue displacements, maintenance of the surgeon’s optical axis at the surgical site without having to look at a navigation monitor, and working freely with both hands without holding the ultrasound scanner during biopsy taking. The described functional model can also be used in open head and neck surgery.

Grob- und Feinnadelbiopsien sind gängige diagnostische Verfahren in der Kopf-Hals-Onkologie. Wegen der Nähe zu lebenswichtigen Strukturen ist eine Punktion von tiefergelegenen Raumforderungen (RF) im weichen Gewebe von Hals oder Parotis schwierig. Hier können Navigationssysteme helfen. Bisher existieren im klinischen Einsatz nur statische Verfahren mit Ungenauigkeiten im Weichgewebe. In diesem Beitrag wird ein ultraschallbasiertes Prinzip vorgestellt und evaluiert. Es zeigt Vorteile im Weichgewebe und gleichzeitig Annehmlichkeiten in der Handhabung.

Eine malignomverdächtige RF sollte histologisch abgeklärt werden. Biopsieentnahmen sind wegen der komplexen Kopf-Hals-Anatomie z. T. gefährlich und bedürfen einer Navigation. Herkömmliche Navigationssysteme sind statische Verfahren, da sie präoperative CT- oder MRT-Daten verwenden [18]. Gewebeverschiebungen während des Eingriffs können nicht dargestellt werden. Darüber hinaus sind sie nur in Beziehung zu starren Landmarken (Knochen) ausführbar. Im Weichgewebe, weit entfernt von knöchernen Strukturen, wird die Navigation aufgrund des „tissue shifts“ ungenau [13]. Deshalb ist der Einsatz dieser Systeme in der Weichteilchirurgie zum Erreichen von kleinen Zielstrukturen ungeeignet. Ziel dieser Arbeit war es, Abhilfe über die Entwicklung eines sonographisch gestützten Verfahrens zu entwickeln, das Gewebeverschiebungen in Echtzeit registriert. Vorteile der Sonographie sind die sofortige Visualisierung von Gewebeveränderungen [5], einfache Handhabung, fehlende Strahlenbelastung, gute Tiefenpenetration und geringe Kosten [19]. Bisherigen ultraschallnavigierten Punktionssystemen lag ein einhändiges Arbeiten zugrunde, da der UK in der anderen Hand gehalten werden musste. Darüber hinaus kommt es zur Störung der optischen Achse des Operateurs, da die Navigationsinformationen auf einem separaten Monitor dargestellt werden [1, 6]. Durch eine automatische Registrierung mittels tiefer neuronaler Netze und durch den Einsatz eines akustischen Feedbacks sollen diese Probleme gelöst werden. Tiefe neuronale Netze als modernes Verfahren maschinellen Lernens haben in den letzten Jahren in der medizinischen Bildanalyse Anwendung gefunden [10, 21].

## Studiendesign und Untersuchungsmethoden

**Abb. 1:**
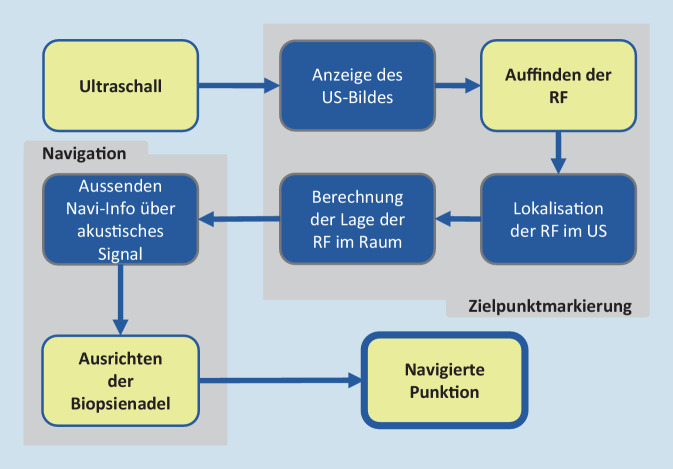
Schematischer Ablauf des Navigationssystems. Erforderliche Schritte durch den Untersucher (*gelb*) und das System (*blau*) sowie die zwei Hauptfunktionen Zielpunktmarkierung und Navigation (*grau*). *US* Ultraschall; *RF* Raumforderung

Das beschriebene Navigationssystems umfasst zwei Hauptabläufe: (1) *Zielpunktmarkierung* und (2) *Navigation* (Abb. 1). (1): Nach sonographischer 2‑D-Einstellung der zu punktierenden RF erfolgt die Registrierung im 3‑D-Raum (2): Einstichstelle, Einstichwinkel und Einstichtiefe werden angezeigt, sodass die Biopsienadel optimal ausgerichtet werden kann. Die Punktion kann beidhändig ohne zusätzliches Halten des UK durchgeführt werden. Der Blick des Operateurs bleibt auf dem Patienten, da die Navigation akustische Rückmeldungen gibt.

### Halsphantom

**Abb. 2:**
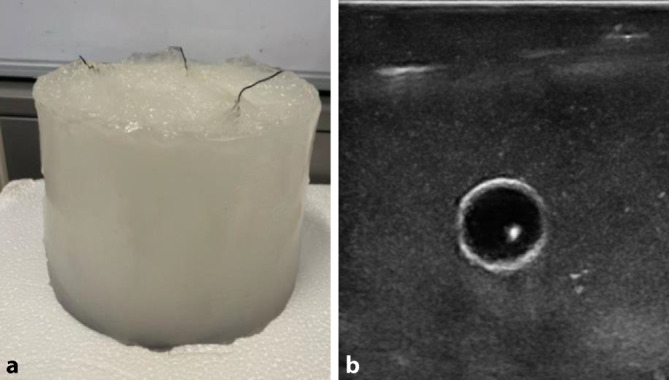
Halsphantom.** a** Gelphantom (12 cm × 10 cm), **b** Ultraschallaufnahme einer artifiziellen Raumforderung im Phantom

Für die Reproduzierbarkeit der Experimente erfolgte die Entwicklung an einem ultraschallfähigem Halsphantom (Abb. 2) angepasst an unsere Segmentierungsanforderungen [7]. Das Modell wurde aus 10 %igem Polyvinyl-Alkohol(PVA)-Gel mit destilliertem Wasser unter Zusatz von Flohsamen hergestellt. Wasserperlen von 8 bis 10 mm Durchmesser wurden als RF eingebracht. Die Größe und die akustischen Eigenschaften entsprechen denen von kleinen pathologischen Halslymphknoten oder kleineren RF in der Parotis. Die Größe wurde sehr klein gewählt, um das System so zu konstruieren, dass auch kleine, schwer zugängliche RF sicher erfasst werden können.

### Ultraschallgerät und Trackingsystem

Als Ultraschallgerät wurde der drahtlose Linearscanner L15 HD3 Wireless Scanner (Fa. Clarius© Mobile Health, Vancouver, Kanada) verwendet [3]. Er bietet die Möglichkeit der Tastenprogrammierung und der Weiterverarbeitung von Bilddaten über ein Application Programming Interface (API). Die Anzeige der Bilder erfolgt auf einem Tablet, welches sich über ein Transmission Control Protocol direkt mit dem Sonographiegerät verbindet.

Anhand der Position der RF im 2‑D-Ultraschallbild wird die Position der RF im globalen 3‑D-Koordinatensystem berechnet (Registrierung). Dafür werden ein Trackingsystem (Polaris Vicra, Fa. Northern Digital Incorporated, Waterloo, Kanada) und eine geeignete Kalibrierungsmethode eingesetzt. Zur Verfolgung des Ultraschallgeräts und der Biopsienadel im Messvolumen wird der UK und die Nadel mit einem Trackingtool versehen. Dafür wurde ein Adapter konstruiert, gedruckt und dem UK aufgesetzt (Form 3B+, SLA-Material Tough 1500 Resin, Fa. Formlabs GmbH, Berlin, Deutschland; Supplement-Abb. 1). Die über das Trackingsystem erfassten Informationen werden an die Navigationsanwendung übergeben. Diese führt auf Grundlage der Cast API des Ultraschallscanners die Zielpunktmarkierung mit anschließender Navigation durch. Das Trackingsystem erfasst die Positionen des Ultraschallgeräts, der RF und der Biopsienadel und sendet diese an die Navigationsanwendung. Die Navigationsanwendung erfolgte Python-basiert (Python-Version: 3.10, Fa. Python Software Foundation, DE, USA) und mit ROS2 Framework Version Humble (Robot Operating System, Fa. Open Source Robotics Foundation, CA, USA).

### Segmentierungsalgorithmus des tiefen neuronalen Netzes

Die Lokalisation der RF im Halsphantom erfordert die Anwendung eines trainierten tiefen neuronalen Netzes. Für die Untersuchungen wurde ein Netz, das auf Brusttumoren mit 697 Scans vortrainiert wurde, verwendet [12]. Die Netzwerkarchitektur beruht auf der Grundlage von ResNet-50.

## Ergebnisse

### Navigation

**Abb. 3:**
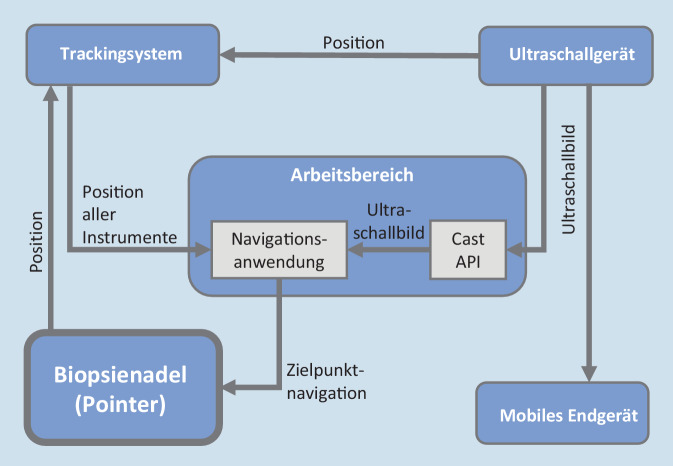
Funktionsmusters des Navigationsablaufs*. Blau *Hardware-Komponenten. *Grau *Software-Komponenten

Die Abb. 3 zeigt den entwickelten Navigationsablauf als Funktionsmuster. Sobald die RF mit dem Sonographiegerät eingestellt ist, betätigt der Operateur eine Taste am UK. Diese wurde zur Weiterverarbeitung der Daten so programmiert, dass damit die Bilddaten an das Trackingsystem gesendet werden. Der UK kann nun ablegt werden, und der Operateur kann beidhändig weiterarbeiten. Im 2‑D-Ultraschallbild wird die RF mittels tiefer neuronaler Netze segmentiert und die Koordinaten des Mittelpunkts der RF berechnet. Anschließend wird eine Transformation der 2‑D-Position im Koordinatensystem des Ultraschallbilds in das 3‑D-Weltkoordinatensystem berechnet. Das Trackingsystem erfasst die Positionen des UK und der Biopsienadel und sendet diese an die Navigationsanwendung. Durch Weiterverarbeitung des Ultraschallbilds und der erfassten Instrumente im Raum wird der Operateur durch die Navigationsanwendung zur RF navigiert. Die Biopsienadel kann an eine beliebige Stelle in der Nähe der RF am Hals angesetzt werden. Um eine optimale Ausrichtung der Nadel zur RF zu erreichen, ist der korrekte *Einstichwinkel* notwendig, für dessen Berechnung Gl. 1 ermittelt wurde. Die berechnete 3‑D-Position der RF legt den Mittelpunkt (*m*) einer Kugel und die Raumkoordinatoren (*x*, *y*, *z*) zugrunde. Wenn die Gerade der Biopsienadel den Mittelpunkt der Kugel schneidet, ist der Winkel korrekt und wird durch ein akustisches Signal bekannt gegeben.

1 $$\begin{aligned}
				&\left(x-m_{x}\right)^{2}+\left(y-m_{y}\right)^{2}+\left(y-m_{y}\right)^{2}\\
				&=r^{2}
				\end{aligned}$$

Für die Berechnung der *Position der RF* (Position RF^Welt^) wurde anhand der Koordinaten der RF im Raum die Gl. 2 entwickelt. Dafür wurde eine Kalibrierung des Ultraschallbilds zum Weltkoordinatensystem erarbeitet. Das Ergebnis der Kalibrierung ist die Transformationsmatrix ^Sono^T_Bild_, das heißt die Transformation T zwischen dem Koordinatensystem des „Bilds“ und dem lokalen Koordinatensystem des am UK befindlichen Trackingtools („Sono“). Aus dem Trackingsystem wurde die Transformationsmatrix ^Welt^T_Sono_ zwischen dem Koordinatensystem des Trackingtools am Ultraschallgerät („Sono“) und dem Weltkoordinatensystem („Welt“) berechnet. Durch Berechnung der Transformationsmatrix ^Welt^T_Bild_ konnte eine 2‑D-Pixelkoordinate (*x*, *y*) der RF des Ultraschallbilds ins Weltkoordinatensystem transformiert werden. Diese gibt multipliziert mit der Position der RF im Bild (Position RF^Bild^) die Lage der RF im Raum (*x*, *y*, *z*) an.

2 $$\begin{aligned}
				\text{Position}\,\textit{RF}^{\text{Welt}}={}&{}^{\text{Welt}}{T}_{\text{Bild}}\\
				&\times\text{Position}\,\textit{RF}^{\text{Bild}}
				\end{aligned}$$

3 $$^{\text{Welt}}{T}_{\text{Bild}}=^{\text{Welt}}{T}_{\text{Sono}}\times^{\text{Sono}}{T}_{\text{Bild}}$$

Zur Realisierung der korrekten Ausrichtung der Biopsienadel wurde eine Berechnung erarbeitet, die es ermöglicht die *Position der Biopsienadelspitze* zu ermitteln. Dafür wurde durch Kalibrierung der Spitze eine Transformationsmatrix ^Nadel^T_Spitze_ zwischen Nadel und Spitze bestimmt. Aus dem Trackingsystem wurde die Transformation zwischen der Nadel und dem Weltkoordinatensystem ^Welt^T_Nadel_ errechnet. Damit ergibt sich die Position der Nadelspitze wie folgt:

4 $$^{\text{Welt}}{T}_{\text{Spitze}}=^{\text{Welt}}{T}_{\text{Nadel}}\times^{\text{Nadel}}{T}_{\text{Spitze}}$$

### Akustisches Feedback und Tiefeninformation

Um ein beidhändiges freies Arbeiten des Operateurs zu ermöglichen, wird, sobald der korrekte Einstichwinkel und die Ausrichtung der Nadel zum Treffen der RF erreicht ist, dies akustisch angezeigt. Nur die entsprechende Eindringtiefe wird zu Beginn als Zahl am Tablet mitgeteilt. Die Positionsberechnung ist von jeglichem Punkt an der Oberfläche des Halses aus möglich. Dafür wird die Biopsienadel aufgesetzt und rotiert. Zu jedem Zeitpunkt einer neu erfassten Position der Biopsienadel werden verschiedene Rückgabewerte gemeldet. Wenn die verlängerte Gerade der Biopsienadel die RF nicht schneiden würde (Rückgabewert von 0), wurde die Ausgabe eines tiefen Tons mit einer Frequenz von 200 Hz erarbeitet. Sobald sich der Operateur durch Rotation des Pointers dem richtigen Winkel nähert (Rückgabewert von 1), wird ein Hinweis durch einen 350-Hz-Ton abgegeben. Wird die Gerade aus Biopsienadel und Nadelspitze die RF treffen (Rückgabewert 2 – optimaler Einstichwinkel), erhält der Operateur die akustische Rückmeldung durch einen hohen 440-Hz-Ton. Die Angaben wurden aus dem quadratischen Abstand zwischen der aktuellen Position der Nadelspitze und der RF berechnet. Da der Operateur die Biopsienadel stetig bewegen können muss, wurde eine Aktualisierungsfunktion der Tiefenberechnung mit einer Aktualisierungsrate von 20 Hz integriert.

### Chirurgische Genauigkeitsmessung mittels euklidischer Distanz im Evaluationsphantom

**Abb. 4:**
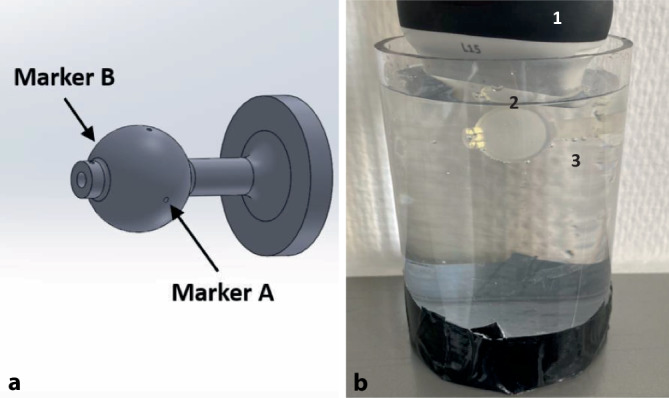
Evaluationsphantom. **a** Computer aided design (CAD)-Modell mit vordefinierten Einkerbungen (Marker A und B) auf der Oberfläche, die auf einer Geraden liegen (Abstand 19 mm). **b** Ausgedrucktes Modell im Wasserbad (Material Elastic 50A, Form 3+, Fa. Formlabs GmbH, Berlin, Deutschland). *1* Clarius Ultraschallscanner, *2* Hohlkugel mit integrierter Raumforderung, *3* Wasserbad

Um die Systemgenauigkeit zu evaluieren, wurde ein Evaluationsphantom entwickelt (Abb. 4), das aus einer wassergefüllten Kugel (Innendurchmesser 16 mm) im Wasserbad mit zwei gegenüberliegenden Einkerbungen (Marker A/B) und einer 8 mm großen RF in der Kugel besteht. Mithilfe des Trackingsystems wurde der Mittelpunkt der Kugel ermittelt. Die Positionen der Einkerbungen und deren Mittelpunkt wurden mit der Biopsienadel getrackt und definieren den „wahren“ Bezugswert. Um die Genauigkeit des Ultraschallnavigationssystems zu bestimmen, wird die Kugel geschallt und die oben beschriebene Zielpunktmarkierung angewandt. Die Auswertung der Positionen ergibt für Marker A die Koordinaten 55.39, 99.97, −1008.40, für B 55.28, 80.19, −1008.90, für den Mittelpunkt der Kugel 55.34, 90.08, −1008.65. Der Wert des Mittelpunkts der RF im Weltkoordinatensystem wird anhand der segmentierten Position im Ultraschallbild über das tiefe neuronale Netz bestimmt. Die Abweichung zwischen dem Messwert und dem wahren Bezugswert ergibt die maximale Abweichung. Der Eigenfehler der Trackingsoftware beträgt herstellungsbedingt 0,25 mm. Die Tab. 1 zeigt die Abweichungen, bestimmt durch die euklidische Distanz. Der mittlere Erwartungswert liegt bei E = 54,8, 91,1, −1006,4. Die maximale chirurgische Abweichung basierend auf der Segmentierung der RF im Ultraschallbild durch das tiefe neuronale Netz und des mit dem Navigationssystem berechneten Mittelpunkts ist 2,5287 mm.

**Tab. 1:** Genauigkeitsbestimmung mittels euklidischer Distanz

Test-Nr.	Position RF^Welt^ (mm)			Abweichung (mm)
	X^a^	Y^a^	Z^a^	
1	53,81	87,90	−1007,23	30,182
2	54,94	92,89	−1006,66	34,664
3	55,97	91,51	−1007,51	19,343
4	54,22	90,32	−1007,28	17,857
5	53,84	89,81	−1006,06	30,052
6	53,72	91,41	−1005,85	34,976
7	55,04	91,03	−1005,63	31,801
8	56,71	90,03	−1007,64	17,028
9	54,41	92,65	−1005,47	41,931
10	55,70	93,18	−1004,92	48,634

*RF* Raumforderung, *Welt* Weltkoordinatensystem

^a^Raumkoordinaten

**Abb. 5:**
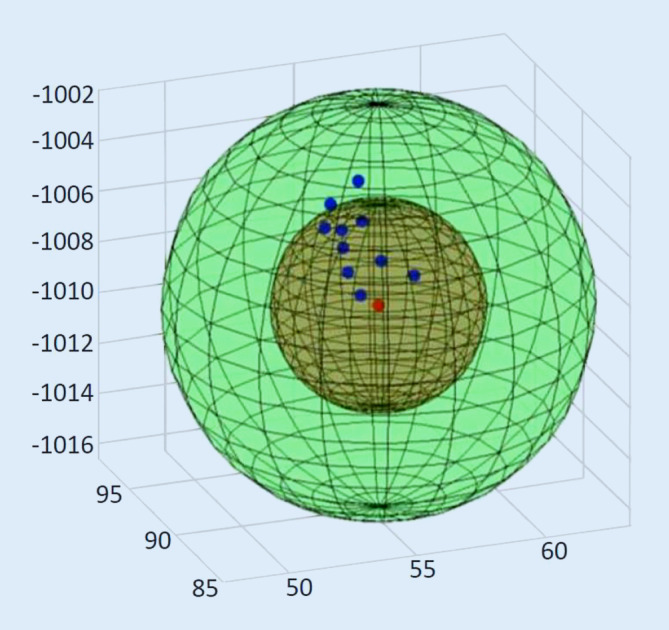
Globusmodell der Hohlkugel. *Grün *Hohlkugel (16 mm Innendurchmesser). *Braun *Raumforderung (8 mm). *Rot *Mittelpunkt. *Blau *navigierte Messwerte

Um die erfassten Messwerte des Experiments in Bezug zum wahren Wert zu setzen, wird ein Globusmodell entwickelt (Abb. 5). Dabei liegen acht von zehn Messwerten innerhalb der RF des Evaluationsphantoms.

## Diskussion

Wir haben einen einfach bedienbaren Prototyp einer Ultraschallnavigation für den Kopf-Hals-Bereich entwickelt. Die Vorteile sind: Echtzeitvisualisierung, Beibehaltung des Blicks des Operateurs auf den Patienten und die Möglichkeit zum freien beidhändigen Punktieren. So können auch RF in der Nähe von sensiblen Strukturen sicherer erreicht werden. Ein Novum ist das akustische Feedback zur Ausrichtung der Biopsienadel. Von jeglichem Punkt der Hautoberfläche können RF navigiert werden. Auf jede Verschiebung des Ziels kann sofort reagiert werden. Gerade der Kopf-Hals-Bereich ist von Weichgewebsverschiebungen („tissue shift“) betroffen, da hier ein knöchernes Bezugsystem fehlt. Herkömmliche Navigationssysteme verwenden präoperative Daten, die intraoperativ entstandene Verschiebungen nicht betrachten. Deshalb können sie für Weichteiloperationen nicht routinemäßig eingesetzt werden. Intraoperative CT- oder MRT-Aufnahmen liefern Momentaufnahmen, sind aber mit hohem Aufwand, Kosten oder Strahlenbelastung verbunden. Da die Sonographie ein echtzeitfähiges Bildgebungsverfahren ist, kann eine Gewebeverschiebung besser berücksichtigt werden. Die Sicherheit der Operation mit sonographischer Navigation kann zusätzlich gesteigert werden. Es wurden auch Hybridsysteme beschrieben, die auf präoperativen Bilddaten basieren und sich mit intraoperativen Ultraschalldaten überlagern, aber den Datensatz nicht stabil aktualisieren können [1]. Helbig et al. entwickelten ein ultraschallgestütztes Navigationssystem, was die Position des chirurgischen Instruments in Echtzeit im Ultraschallmonitor anzeigt [6]. Nachteilig an diesem System ist sowohl der Wechsel der optischen Achse zwischen Monitor und Patient als auch das einhändige Arbeiten, da der UK in der anderen Hand gehalten werden muss. Ein Wechsel der chirurgischen Blickachse reduziert die intuitive Durchführung des Eingriffs, woraus Fehler und längere Op.-Zeiten folgen [20]. Das in der vorliegenden Arbeit entwickelte Funktionsmuster schafft Abhilfe, indem die wesentlichen Navigationsinformationen für eine Biopsie (Einstichwinkel und Ausrichtung der Biopsienadel) akustisch angegeben werden. Durch die Segmentierung über tiefe neuronale Netze kann der UK nach Einstellen der Zielstruktur abgelegt werden. Der Operateur ist während des Ausrichtens der Biopsienadel frei und kann beidhändig weiterarbeiten.

Neuere Entwicklungen verwenden Augmented-Reality-Systeme, um Ultraschallbilder virtuell in ein auf dem Kopf getragenes System (Head-Mounted-Display) zu projizieren. Maas et al. beschreiben eine virtuelle Darstellung einer Nadel im Ultraschallbild, über die eine Biopsie erleichtert wird, da der Anwender den Blick auf den Operationsbereich fokussieren kann [15]. Augmented-Reality-Systeme können auch bei der Navigation in der offenen Kopf-Hals-Chirurgie helfen, die optische Achse auf den Op.-Situs beizubehalten [16, 17].

Neben dem Einsatz von Augmented Reality kommt auch die Nutzung von neuronalen Netzen in der chirurgischen Navigation zur Anwendung, da hiermit sehr genau automatisch segmentiert werden kann [2, 22]. Als modernes Verfahren maschinellen Lernens modellieren sie abstrakte Daten durch lineare und nichtlineare Verarbeitungseinheiten, die in einer tiefen Architektur angeordnet sind [4]. Nachteilig ist die große Datenmenge zum Training des Netzes, die in der Medizin nur begrenzt verfügbar ist. Alternativ können deshalb semiautomatische Algorithmen zur Segmentierung einer Zielstruktur eingesetzt werden. Beispiele für diese Verfahren sind kontur- und formbasierte [9] oder regionenbasierte Methoden [14]. Diese Verfahren sind zeitaufwendig und weisen einen hohen untersucherabhängigen Fehler auf. Deshalb, und auch um die Trainingsdaten für neuronale Netze zu reduzieren, wurde in dieser Stude die Lokalisierung der RF im Ultraschallbild über ein vortrainiertes tiefes neuronales Netz realisiert („transfer learning“). Dieses erreicht auch hohe Segmentierungsgenauigkeiten [11].

Eine Besonderheit der vorliegenden Studie ist die Genauigkeitsmessung. Dafür wurden ideale Randbedingungen geschaffen, indem ein eigenes Evaluationsphantom mit definierter Geometrie entwickelt wurde. Durch Einbringen des Modells in Wasser wurde eine Eigendeformation verhindert. Die Messergebnisse zeigen, dass das Navigationssystem eine chirurgische Genauigkeit von 2,5 mm liefert. Die Messwerte in der x‑ und z‑Dimension streuen um den Mittelwert ca. 1 mm, in y‑Richtung um 1,6 mm. Um die echte Genauigkeit zu bewerten, müssen alle Unsicherheiten betrachtet werden. Die mit der Biopsienadelspitze erfasste Geometrie der RF zeigt eine Abweichung von 0,79 mm. Der Eigenfehler des Trackingsystems fließt mit 0,25 mm ein. Es kann abgeleitet werden, dass trotz einer Abweichung des jeweiligen Messwerts zum wahren Wert alle Messwerte innerhalb der RF im Evaluationsphantom liegen. Repräsentiert die RF eine Zielstruktur im Halsphantom von nur 8 mm, bedeutet dies, dass sie von der Biopsienadel in acht von zehn Fällen getroffen wird. Mittelfristig sollte eine Verbesserung der Systemgenauigkeit erzielt werden. Dies kann durch Optimierung der Kalibrierung erreicht werden. Ein direkter Vergleich mit anderen Navigationssystemen ist nicht möglich, da verschiedene Randbedingungen vorliegen. Grundlegender Unterschied ist, dass traditionelle Navigationssysteme auf präoperativen Daten basieren, die intraoperativ registriert werden. Dem hier angewendeten Funktionsmuster liegt der Einsatz von intraoperativen Ultraschalldaten zugrunde, die in Echtzeit während des Eingriffs kalibriert werden. In der Literatur sind verschiedene Methoden zur Kalibrierung eines Ultraschallgeräts zu finden. Die Großzahl basiert auf der Verwendung von ultraschallfähigen Phantomen mit bekannter Geometrie und eines Trackingtools am Ultraschallgerät [8], was auch hier verwendet wurde.

## Limitationen

Um den Prototyp klinisch einsetzen zu können, wären folgende Änderungen hinsichtlich des Studiendesigns notwendig:

- Vergrößerung der Anzahl an Probebiopsien
- Anwendung des Systems im Kadavermodell und in vivo
- Optimierung des Kalibrierungsverfahrens mit Erhöhung der Gesamtgenauigkeit auf < 2 mm
- Anpassung der Trainingsdaten des tiefen neuronalen Netzes an Daten von Kopf-Hals-Tumoren

## Ausblick

Das bestehende Funktionsmuster soll in einer klinischen Erprobung weiterentwickelt werden. Die bisherigen Ergebnisse zeigen, dass ein ergonomischer Ultraschallnavigationsprozess technisch realisierbar ist. Das neuronale Netz wird im nächsten Schritt noch spezifischer auf die Kopf-Hals-Anatomie trainiert. Versuche eines 3‑D-Projektionsscanners, der Navigationsinformationen direkt auf den Situs projiziert, sind in unserer Arbeitsgruppe im Entwicklungsprozess. Darüber hinaus werden aktuell zwei weitere Trackingverfahren untersucht, die eine höhere Genauigkeit aufweisen.

## Fazit für die Praxis

- Die Arbeit zeigt den Prototyp eines Ultraschallnavigationssystems, mit dem kleine RF zielsicher punktiert werden können.
- Durch die Verwendung von tiefen neuronalen Netzen und die Entwicklung eines akustischen Feedbacks ergeben sich folgende Vorteile:
  Verzicht auf eine präoperative Bildgebung,
  automatische Echtzeitregistrierung, die intraoperative Gewebeverschiebungen berücksichtigt,
  Beibehaltung der optischen Achse des Operateurs auf den Situs, ohne Blickwechsel auf einen Navigationsmonitor,
  beidhändiges Arbeiten ohne Halten des UK während der Punktion.
- Möglichkeiten und Grenzen des Systems wurden aufgezeigt und Optimierungsvorschläge erarbeitet.

### Supplementary Information

Abb. S1. 3‑D-Adapter für das Ultraschallgerät. Der Adapter befestigt das Trackingtool am Ultraschallgerät. A: CAD-Modell. B: ausgedruckter Adapter mit Navigationstool. Der Adapter wird an die Geometrie des Ultraschallgeräts angepasst. Er ist abnehmbar und sterilisierbar.
