# Erratum zu: Entwicklung und Evaluation einer Ultraschallnavigation für Freihandbiopsien kleiner Raumforderungen im Kopf-Hals-Bereich

**DOI:** 10.1007/s00106-024-01455-6

**Published:** 2024-04-11

**Authors:** Claudia Scherl, Marie Otto, Ibrahim Ghanem, Javier Moviglia, Fabian Sadi, Tirza Gnilka, Nicole Rotter, Lena Zaubitzer, Jan Stallkamp

**Affiliations:** 1 Klinik für Hals-Nasen-Ohrenheilkunde, Kopf- und Halschirurgie, Medizinische Fakultät Mannheim, Universität Heidelberg, Theodor-Kutzer-Ufer 1–3, 68167 Mannheim, Deutschland; 2 AI Health Innovation Cluster, Heidelberg-Mannheim Health and Life Science Alliance, Heidelberg, Deutschland; 3 Mannheim Institute for Intelligent Systems in Medicine (MIISM), Medizinische Fakultät Mannheim, Universität Heidelberg, Heidelberg, Deutschland

**Erratum zu:**

**HNO 2023**

10.1007/s00106-023-01385-9

In diesem Artikel wurde der Name von dem Autor Javier Moviglia fälschlicherweise als Javier Oviglia angegeben.

Der Originalbeitrag wurde korrigiert.

