# Supplementary material for: Development and evaluation of ultrasound navigation for free-hand biopsies of small masses in the head and neck area
Source: HNO. 2023 Dec 5;72(2):76–82. [Article in German] doi: 10.1007/s00106-023-01385-9 (PMC11362245; doi:10.1007/s00106-023-01385-9)

Abb. S1 3D-Adapter für das Ultraschallgerät.

Der Adapter befestigt das Trackingtool am Ultraschallgerät. A: CAD-Modell. B: ausgedruckter Adapter mit Navigationstool. Der Adapter wird an die Geometrie des Ultraschallgerätes angepasst. Er ist abnehmbar und sterilisierbar.

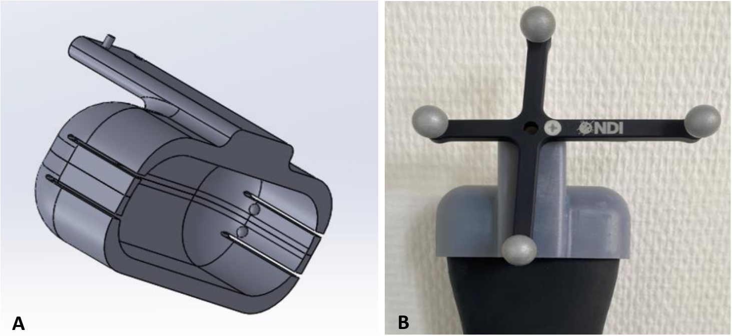

Supplement: Supplementary file 1 — Abb. S1. 3‑D-Adapter für das Ultraschallgerät. Der Adapter befestigt das Trackingtool am Ultraschallgerät. A: CAD-Modell. B: ausgedruckter Adapter mit Navigationstool. Der Adapter wird an die Geometrie des Ultraschallgeräts angepasst. Er ist abnehmbar und sterilisierbar. [file 106_2023_1385_MOESM1_ESM.pdf]
